# C6-Ceramide Nanoliposomes Target the Warburg Effect in Chronic Lymphocytic Leukemia

**DOI:** 10.1371/journal.pone.0084648

**Published:** 2013-12-19

**Authors:** Lindsay K. Ryland, Ushma A. Doshi, Sriram S. Shanmugavelandy, Todd E. Fox, Cesar Aliaga, Kathleen Broeg, Kendall Thomas Baab, Megan Young, Osman Khan, Jeremy K. Haakenson, Nancy Ruth Jarbadan, Jason Liao, Hong-Gang Wang, David J. Feith, Thomas P. Loughran Jr, Xin Liu, Mark Kester

**Affiliations:** 1 Department of Pharmacology, Penn State College of Medicine, Hershey, Pennsylvania, United States of America; 2 Penn State Hershey Cancer Institute, Penn State College of Medicine, Hershey, Pennsylvania, United States of America; 3 Department of Medicine, Penn State College of Medicine, Hershey, Pennsylvania, United States of America; Medical University of South Carolina, United States of America

## Abstract

Ceramide is a sphingolipid metabolite that induces cancer cell death. When C6-ceramide is encapsulated in a nanoliposome bilayer formulation, cell death is selectively induced in tumor models. However, the mechanism underlying this selectivity is unknown. As most tumors exhibit a preferential switch to glycolysis, as described in the “Warburg effect”, we hypothesize that ceramide nanoliposomes selectively target this glycolytic pathway in cancer. We utilize chronic lymphocytic leukemia (CLL) as a cancer model, which has an increased dependency on glycolysis. In CLL cells, we demonstrate that C6-ceramide nanoliposomes, but not control nanoliposomes, induce caspase 3/7-independent necrotic cell death. Nanoliposomal ceramide inhibits both the RNA and protein expression of GAPDH, an enzyme in the glycolytic pathway, which is overexpressed in CLL. To confirm that ceramide targets GAPDH, we demonstrate that downregulation of GAPDH potentiates the decrease in ATP after ceramide treatment and exogenous pyruvate treatment as well as GAPDH overexpression partially rescues ceramide-induced necrosis. Finally, an *in vivo* murine model of CLL shows that nanoliposomal C6-ceramide treatment elicits tumor regression, concomitant with GAPDH downregulation. We conclude that selective inhibition of the glycolytic pathway in CLL cells with nanoliposomal C6-ceramide could potentially be an effective therapy for leukemia by targeting the Warburg effect.

## Introduction

Sphingolipids are a class of complex cellular lipids that serve both a structural role in the cellular membrane as well as an intracellular signaling role within the cell. Several types of sphingolipid metabolites have been shown to influence the balance between mitogenesis and apoptosis. Of particular interest is the sphingolipid metabolite, ceramide, which is known to regulate differentiation, senescence and cell cycle arrest. Induction of cell death by this endogenous lipid-derived second messenger occurs either via apoptotic, autophagic, or necrotic cell death pathways [1,2,3]. Ceramide inhibits cell proliferation and induces apoptosis via mechanisms such as dephosphorylation and/or inactivation of molecules including Akt, phospholipase D, ERK, Bcl-2, survivin, PKC-α, and pRB [4,5,6], as well as activation of JNK kinases[4,7], or PKC zeta which, results in suppression of Akt-dependent mitogenesis [8]. Therefore, it is not surprising that dysregulated ceramide metabolism and signaling has been linked to a variety of human diseases, including cancer. Based on its ability to selectively block tumor initiation and metastasis, ceramide has been termed the ‘tumor-suppressor lipid’ [4]. Many cancer chemotherapies have been shown to generate endogenous ceramide, and when *de novo* generation of ceramide is inhibited, the cellular response to cytotoxic chemotherapeutic agents decreases [4]. In addition, it has previously been shown that accumulation of endogenous ceramides or exogenous ceramide treatment is more toxic to tumor cells than to normal cells [6,9]. However, the exact mechanism of selectivity is unknown. 

One proposed mechanism for how ceramide mediates cell death induction is through downregulation of nutrient transporter proteins possibly via nutrient deprivation [10].. As cancer cells have an increased dependence on glucose, these nutrient transporters and/or the glycolytic pathway are typically upregulated. One hallmark of cancer cells is their ability to avidly take up glucose and convert it to lactate, even in the presence of sufficient oxygen. Deemed the “Warburg effect,” this altered glycolytic dependency favors less efficient generation of ATP compared to the oxidative phosphorylation process which occurs in normal cells [11,12]. Many human cancers display increased levels of glycolytic enzymes compared to normal tissue [13]. Consequently, a variety of chemotherapeutic glycolytic inhibitors or PET modalities are currently under investigation as potential “Warburg-targeted” therapeutic or diagnostic imaging tools [14,15]. Recently, the role of sphingosine kinases in regulating the Warburg effect in prostate cancer cells has been documented in the literature [16]. Treatment of LNCaP prostate cancer cells with SKi, a non-selective sphingosine kinase inhibitor, significantly increases intracellular levels of ceramide and sphingosine and indirectly antagonizes the Warburg effect, resulting in apoptosis of LNCaP cells. 

Chronic lymphocytic leukemia (CLL) is the most common B-cell malignancy in the Western world which presently has no known curative therapy [17]. Previous studies have demonstrated that treatment with exogenous short-chain C2-ceramide results in induction of cell death in malignant cells isolated from CLL patients [18]. Recent advances in nanotechnology have illustrated the feasibility of generating nanoliposomes that encapsulate hydrophobic compounds, like ceramide, to facilitate treatment of CLL. While it is understood how nanoliposomal ceramide induces cell death in several types of cancers and hematological malignancies, the effect of nanoliposomal ceramide treatment in CLL remains unclear. Currently, several nanoliposomal formulations of anti-cancer drugs have been approved by the FDA and are the standard of care [19]. For instance, the efficacy of fludarabine, the cancer chemotherapy commonly used to treat CLL patients, and which acts via intracellular ceramide accumulation, is enhanced after being encapsulated in nanoliposomes [20,21]. Our laboratory has demonstrated that encapsulation of ceramide in a nanoliposome versus non-liposomal organic formulations results in an increase in cytotoxic potential with significant less toxicity [22]. Our laboratory has also demonstrated that the short chain C_6_-ceramide nanoliposomal formulation displays anti-proliferative effects *in vitro*, as well as results in tumor regression in several animal models of cancer [6,9,22,23]. 

In this study we sought to elucidate the effect of nanoliposomal C6-ceramide treatment in CLL. Our data suggest that this treatment is targeting glucose utilization and results in activation of a caspase-independent, necrotic cell death mechanism. We have identified glyceraldehyde 3-phosphate dehydrogenase (GAPDH) as a novel target of ceramide in CLL cells. In the current study, we conclude that ceramide targets the Warburg effect in cancer cells and selectively induces necrotic cell death in CLL. 

## Methods

### Reagents

Antibodies specific for caspase 3, poly ADP ribose polymerase (PARP), GAPDH, β-actin and α-tubulin were purchased from Cell Signaling Technology Inc. (Beverly, MA), glucose transporter 1 (GLUT1) antibody from Abcam (Cambridge, MA) and lactate degydrogenase (LDH) antibody from Epitomics (Burlingame, CA). For Western blotting, 12% precasted Nupage electrophoresis gels from Invitrogen (Carlsbad, CA), and chemiluminescence reagent from Amersham Biosciences Inc. (Piscataway, NJ) were obtained. Other reagents include zVAD-fmk and pyruvate from Sigma (St. Louis, MO), dasatinib from Toronto Research Chemicals Inc. (Ontario, Canada) and 3-bromopyruvate from Enzo Life Sciences (Farmingdale, NY). 

### Patient characteristics and preparation of peripheral blood mononuclear cells

All patients met the clinical criteria of CLL and were not on treatment at the time of sample acquisition. Peripheral blood specimens from CLL patients were obtained and informed consents signed for sample collection using a protocol approved by the Institutional Review Board of Penn State Hershey Cancer Institute. Buffy coats from normal donors were also obtained from the blood bank of the Milton S. Hershey Medical Center, Pennsylvania State University, College of Medicine. Peripheral blood mononuclear cells (PBMCs) were isolated by Ficoll-Hypaque gradient separation, as described previously [24]. Cell viability was determined by trypan blue exclusion assay with more than 95% viability in all the samples. 

### Cell culture

Freshly isolated PBMCs and primary CLL patient cells were cultured using RPMI-1640 medium supplemented with 10% fetal bovine serum (both from Invitrogen). JVM3 cells (DSMZ – German Collection of Microorganisms and Cell Cultures, Braunschweig, Germany), a CLL cell line, were also cultured in this same medium and cells were grown in 5% CO_2_ at 37°C.

### Preparation of nanoliposomal ceramide

12% pegylated nanoliposomes (80 ± 15 nm in size) that contain 30 mol% ceramide were prepared as described previously with lipids 1,2-distearoyl-sn-glycero-3-phosphocholine, 1,2-dioleoyl-*sn*-glycero-3-phosphoethanolamine, *N*-hexanoyl-d-erythro-sphingosine (C_6_-ceramide), 1,2-distearoyl-sn-glycero-3-phosphoethanolamine-*N*-[methoxy polyethylene glycol-2000], and *N*-octanoyl-sphingosine-1-[succinyl(methoxy polyethylene glycol-750)] (PEG(750)-C_8_) combined in chloroform at a molar ratio of 3.75:1.75:3:0.75:0.75 [22]. Combined lipids were dried under nitrogen gas and resuspended in 0.9% sterile NaCl at 60°C. Following rehydration, resulting solution was sonicated for 5 min followed by extrusion through a 100-nm polycarbonate membrane using the Avanti Mini Extruder (Avanti Polar Lipids). Ghost liposomes were prepared in a similar manner excluding *N*-hexanoyl-d-erythro-sphingosine (C_6_). Dihydro-C6-ceramide liposomes were prepared in a similar manner by replacing *N*-hexanoyl-d-erythro-sphingosine with N-hexanoyl-D-erythro-sphinganine. Several QA/QC parameters were evaluated after preparation of nanoliposomes. Nanoliposomes were formulated within the size range of 85nM - 90nM as measured by dynamic light scattering. Zeta potentials of the nanoliposomes were measured and these were between -3 mV to -7 mV, ensuring a neutral charge on the nanoliposomes. Encapsulation efficiency of the ceramide nanoliposomes was evaluated by LC/MS/MS. 

### Cell viability assay

Cell viability was performed using CellTiter 96® Aq_ueous_ One Solution assay kit (Promega) or alamarBlue® assay kit (Invitrogen). Relative viable cell number was determined by reading the plates at 490 nm or 570 nm wavelength respectively in Synergy HT Multi-Detection Microplate Reader (Bio-TEK). All samples were assayed in triplicate and each experiment was repeated at least three times. 

### Apoptosis and exclusion assays (Annexin V/7AAD and TUNEL)

Apoptosis was determined in JVM3 cells and in PBMC samples from normal donors (n=3) by 2-color flow cytometry with annexin-V and 7-amino-actinomycin D (BD Pharmingen Transduction Laboratories) staining using 5 x 10^5^ cells per sample. TUNEL assays were also performed using terminal deoxylnucleotidyl transferase (TdT), biotinylated UTP, and fluorescein isothiocyanate-streptavidin as described previously [25]. Cell viability was also confirmed by trypan blue exclusion. 

### Caspase 3/7 assay

For detection of caspase-3 and caspase-7 activation, JVM3 cells were plated in replicates of six in 96-well plates, and treated with varying doses of ceramide for 24 hours and analyzed using ApoONE Homogeneous Caspase 3/7 Assay (Promega) according to the manufacturer's instructions. 

### Western blot analysis

Western blot analyses of caspase 3, PARP, GAPDH, LDH, GLUT1, α-tubulin and β-actin protein expression were performed on whole-cell lysates collected using RIPA buffer (Sigma). Densitometry analysis was performed using ImageJ software. 

### Phase-contrast microscopy

To visualize a morphological necrotic phenotype, JVM3 cells were plated at 1 x 10^6^ cells/ well and treated with 25 µM ghost nanoliposomes for 24 hours or C6-ceramide nanoliposomes for 2, 6 and 24 hours. Phase contrast microscopy images were then taken (Olympus CKX41).

### GAPDH gene expression: real-time quantitative RT-PCR

Real-time reverse-transcription polymerase chain reaction (RT-PCR) was performed using primer sets specific for GAPDH and an internal standard, 18S rRNA, in an ABI PRISM 7900 sequence detector (Applied Biosystems) as described elsewhere [6]. TRIzoL LS Reagent (Invitrogen) was used for RNA extraction. Amplification of triplicate cDNA template samples was performed with denaturation for 15 minutes at 95°C, followed by 45 PCR cycles of denaturation at 94°C for 15 seconds, annealing at 55°C for 30 seconds, and extension at 72°C for 30 seconds. A standard curve of cycle thresholds using serial dilutions of cDNA samples was established and used to calculate the relative abundance of the target gene. Values were normalized to the relative amount of 18S mRNA. The relative amount of PCR products generated from each primer set was determined based on the threshold cycle or threshold cycle value [26]. The following primers were used for detection: GAPDH sense 5’-GACCCCTTCATTGACCT CAACTACATG -3’, GAPDH antisense 5’- GTCCACCACCCTGTTGCTGTAGCC-3’.

### Glucose uptake and lactate production

The [^3^H]-2-DG uptake assay was modified from Kueck et al. [27]. 2-[1,2,-^3^H (N)]-Deoxy-D-glucose was purchased from PerkinElmer. Cells were plated in 24-well plates and 24 hours after experimental treatment were washed with PBS and resuspended in Krebs-Ringer phosphate buffer + 1% BSA. Glucose uptake was initiated by adding 1 µCi/mL ^3^H-2DG and 10 mM unlabeled glucose for 15 minutes. Uptake was terminated by adding ice cold Krebs-Ringer phosphate buffer + 0.2 mM phloretin. 20 µM cytochalasin B, a potent inhibitor of glucose transport was used as a control. All experiments were done in triplicate. Lactate concentrations in culture media were determined after nanoliposomal treatment using a colorimetric assay kit (Biomedical Research Service Center, SUNY at Buffalo). 

### Measurement of ATP

Cellular ATP content was measured using a luminescence assay (Cell-Titer Glo Kit, Promega). JVM3 cells were pretreated in the absence or presence of pyruvate for 2 hours, and then incubated for 24 hours with nanoliposomal ceramide. Final luminescence was measured in Synergy HT Multi-Detection Microplate Reader (Bio-TEK). 

### shRNA Knockdown of GAPDH

GAPDH shRNA lentiviral clones (Human pLKO.1 vector) were purchased from Open Biosystems (Huntsville, AL) and used to infect JVM3 cells according to the manufacturer’s protocol. After selection with puromycin, a pool of infected cells were expanded and tested for GAPDH expression via Western blotting. Scrambled shRNA was used as a control in these experiments.

### Lentiviral overexpression of GAPDH

Human pLOC vector (Open Biosystems) was used for overexpressing GAPDH in JVM3 cells. Briefly, viral particles were produced in HEK293-FT cells using pLOC, VSVG, tat and DR8.2 plasmids and JVM3 cells were transduced thrice with the viral media according to the manufacturer’s protocol. JVM3 cells were grown for 72 hours after the last transduction and subsequently harvested for experiments. GAPDH overexpression was detected 72 hours after last transduction via Western blotting. pLOC vector containing a red fluorescent protein (RFP) sequence instead of GAPDH gene was used as a control in these expreiments. 

### Animal Studies

Animal experimentation was performed according to protocols approved by the Institutional Animal Care and Use Committee at the Pennsylvania State College of Medicine and all efforts were made to minimize animal suffering. Female Balb/c Nu/nu mice of about six weeks of age were obtained from Charles River Laboratory (Wilmington, MA). Mice were subject to irradiation (600 cGy) one day prior to inoculation. Ten million JVM3 cells were subcutaneously injected into the right flank of the mice and treatment began approximately two weeks after inoculation when tumors reached a volume of 50 to 100 mm^3^. Based upon prior studies with multiple animal models [6,9,22,23], leukemic mice were treated with 40 mg/kg ghost (n=8) or C6-ceramide nanoliposomes (n=8) via IV injection every other day over a three week period of time. Mice with large or ulcerated tumors were euthanized.

In a parallel study, leukemic mice treated with an identical dose regimen of ghost or C6-ceramide nanoliposomes were sacrificed during the course of the study and tumor tissue was then flash frozen and isolated for protein extraction. Mice were sacrificed on day 8 of C6-ceramide treatment (n=5), day 14 of C6-ceramide treatment (n=6) and day 17 of C6-ceramide treatment (n=6). Additionally, mice treated with the ghost nanoliposomes for 17 days were also sacrificed (n=5) and protein was extracted from the tumor tissue for further immunoblot analysis.

### Ethics Statement

A protocol approved by the Institutional Review Board of Penn State Hershey Cancer Institute (Protocol #29839) was used to collect peripheral blood specimens from CLL patients. Animal experiments were performed according to a protocol (Protocol #2009-017) approved by the Institutional Animal Care and Use Committee at the Pennsylvania State College of Medicine and all efforts were made to minimize animal suffering.

### Statistical analysis

All data are expressed as mean +/- SEM. All the graphs represent at least three independent experiments, each replicated in triplicate, unless specified otherwise. Paired Student *t* test (2-tail paired) and 2 way analysis of variance test were used to determine the statistical significance and *P* value of </= 0.05 was considered statistically significant. 

## Results

### Nanoliposomal C6-ceramide selectively induces cell death in CLL cells

**Figure 1:**
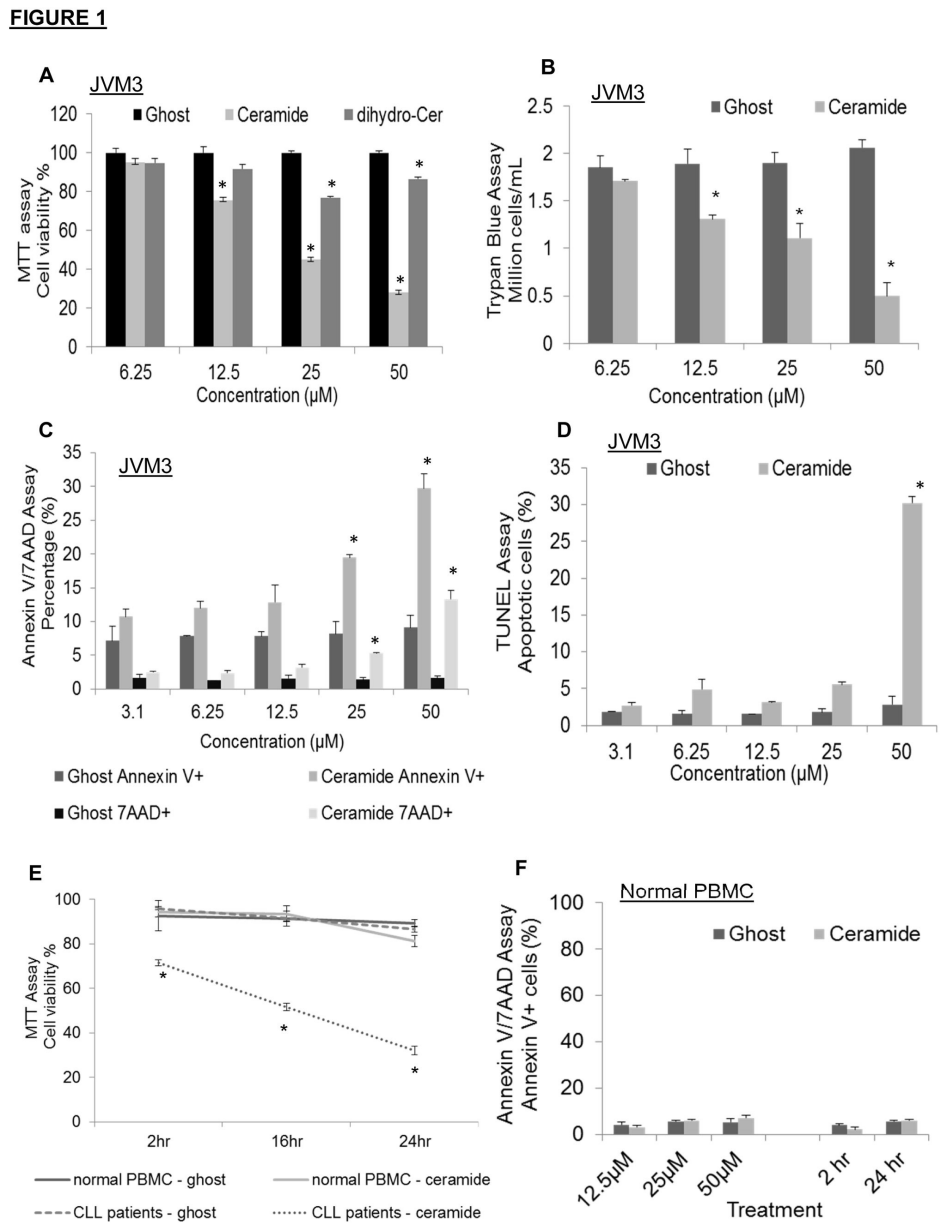
Nanoliposomal C6-ceramide selectively induces cell death in CLL cells. JVM3 cells were treated with varying doses of ghost or C6-ceramide or dihydro-C6-ceramide nanoliposomes for 24 hours then A). MTT assay, B). Trypan blue staining was performed. ANOVA statistical test was used to determine dose dependency between various C6-ceramide treatment groups. *P* < .0001. C). JVM3 cells were treated with different doses of ghost or C6-ceramide nanoliposomes for 24 hours, then cells were stained with annexin V and 7AAD for apoptosis assay. D). Percentage of apoptotic cells was determined via TUNEL analysis after 24 hours. E). PBMC isolated from either CLL patients (n=3) or normal donors (n=3) were treated with 25 µM ghost or C6-ceramide nanoliposome for 2, 16 and 24 hours, then MTT assay was performed. F). Percentage of apoptotic cells was determined in PBMC from normal donors (n=3) via annexin V/7AAD staining. Cells were treated with different doses of ghost or C6-ceramide nanoliposome for 24 hours or treated with 25 µM ghost or C6-ceramide nanoliposome for 2 and 24 hours. * *P* < 0.05.

We have previously demonstrated the therapeutic use of C6-ceramide nanoliposomes in both solid and non-solid tumor models [6,9,22,23]. In the present study, we investigated the therapeutic efficacy of this nanoliposomal formulation in CLL. The CLL *in vitro* JVM3 cell line utilized for these studies was established by EBV-transformation of human primary B-prolymphocytic leukemic cells and treatment with phorbol ester, TPA [28]. MTT assay (Figure 1A) and trypan blue staining (Figure 1B) demonstrated that nanoliposomal C6-ceramide, but not the ghost nanoliposomes, induced dose-dependent cell death in JVM3 cells. Dihydro-C6-ceramide, a less active analog of C6-ceramide modestly reduced cell viability at higher doses (Figure 1A); however C6-ceramide was significantly more toxic to cells in comparison. Concentrations of C6-ceramide nanoliposomes above 25µM significantly increased percentage of non-viable cells as shown by annexin V/7AAD staining (Figure 1C) and TUNEL flow cytometry analysis (Figure 1D). It was further demonstrated that this dose-dependent cell death induced by nanoliposomal C6-ceramide was also observed in primary CLL patient cells but not in PBMC isolated from normal donors (Figure 1E). Flow cytometry analysis confirmed that apoptosis was not induced in these normal cells (Figure 1F). These results indicate that nanoliposomal C6-ceramide is preferentially targeting CLL cells and is non-toxic to normal donor cells. 

### Cell death induced by nanoliposomal C6-ceramide is independent of caspase 3/7

**Figure 2:**
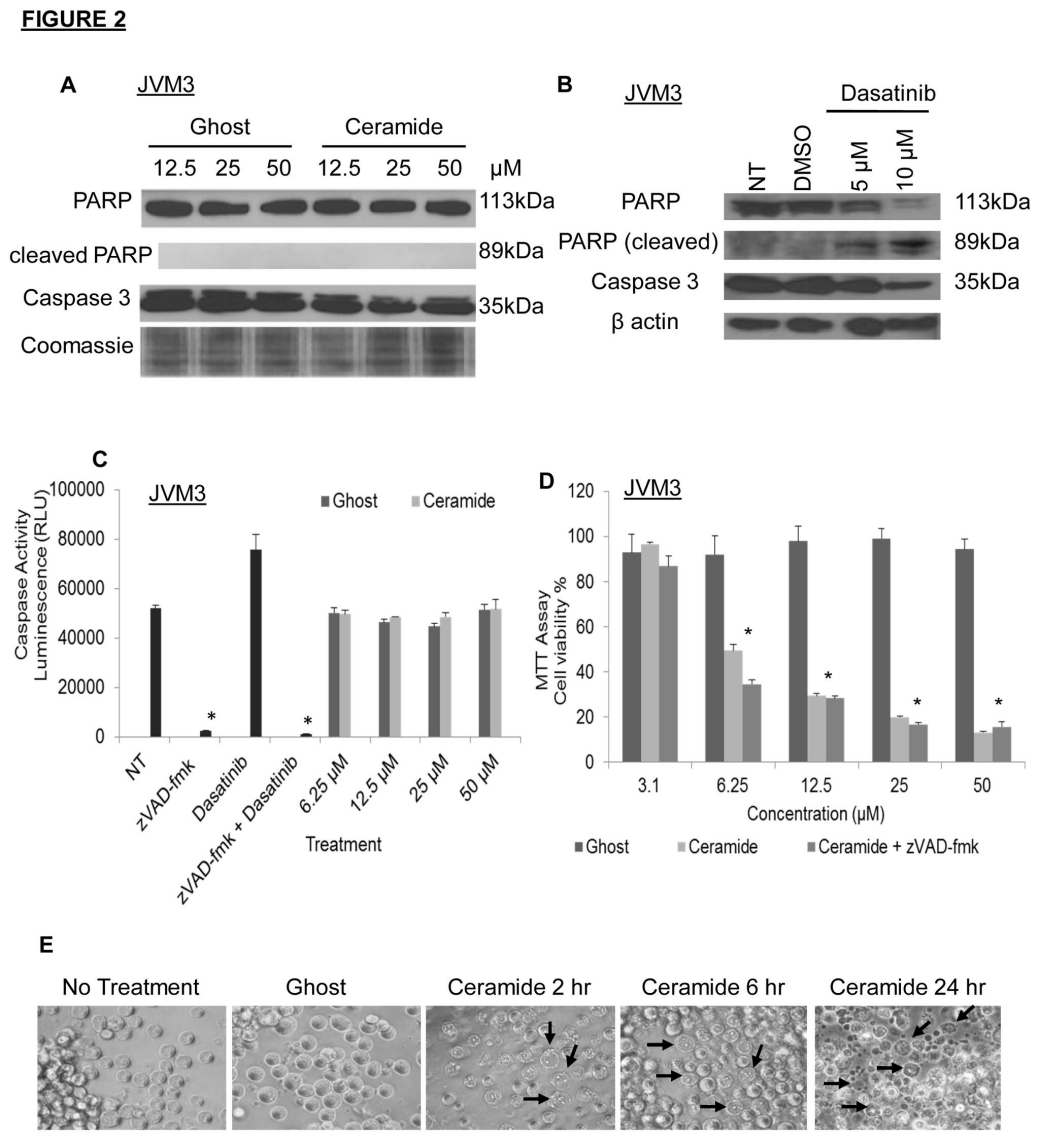
Cell death induced by nanoliposomal C6-ceramide occurs through caspase 3/7-independent necrosis. JVM3 cells were treated with A). different doses of ghost and C6-ceramide nanoliposome for 24 hours, B) 5 µM and 10 µM dasatinib, as well as DMSO vehicle control for 24 hours, then Western Blot analysis was performed for caspase 3 and PARP cleavage. C). Enzymatic activities of caspase 3/7 were measured using caspase 3/7 luminescence kit. * *P* < 0.05. D). JVM3 cells were treated with varying doses of ghost or C6-ceramide nanoliposomes for 24 hours following a 2 hour pre-treatment or no pre-treatment with zVAD-fmk (15 µM). Cell viability was assessed via MTT assay. * *P* < 0.05. E). JVM3 cells were treated with 25 µM ghost nanoliposomes for 24 hours or C6-ceramide nanoliposomes for 2, 6 and 24 hours and phase contrast microscopy images were taken. Arrows indicate morphology of necrotic cell death.

To further characterize the mechanism of cell death being induced in the JVM3 cell line, we investigated the effect of nanoliposomal C6-ceramide treatment on caspase cleavage. Caspase 3 or downstream PARP cleavage was not observed following treatment with the C6-ceramide nanoliposomes (Figure 2A). Treatment of JVM3 cells with 5 and 10 µM dasatinib, an inducer of apoptosis in CLL [29], caused cleavage of both caspase 3 and PARP (Figure 2B), thus confirming that there was no defect in the caspase 3/7 apoptotic pathway in this cell line. No change in caspase activity was also confirmed by the caspase 3/7 assay (Figure 2C). As controls, 5µM dasatanib was sufficient to stimulate caspase activity and pretreatment with 15µM zVAD-fmk, a pan-caspase inhibitor, inhibited this activation. We further confirmed that C6-ceramide-induced cell death was independent of caspase activation, as pre-treatment with zVAD-fmk did not rescue cell death (Figure 2D). 

We next investigated if this caspase 3/7-independent cell death results in a necrotic phenotype. To determine the predominant cell death mechanism being induced following nanoliposomal ceramide treatment, JVM3 cells were treated with 25 µM ghost or C6-ceramide nanoliposomes for varying time points and visualized using phase contrast microscopy (Figure 2E). Arrows indicate cells which have increased in cellular volume and resemble a necrotic morphology as evidenced by early plasma membrane rupture and abundance of cellular debris. In addition, we confirmed necrotic cell death by flow cytometric analysis and observed a time-dependent increase in Annexin V positive and 7AAD positive cell population after treatment with nanoliposomal ceramide for 24 hours (data not shown). From these results, we conclude that the preferential selectivity of ceramide treatment appears to target a necrotic cell death mechanism, consistent with caspase 3/7-independent cell death. 

### GAPDH as a target for treatment of CLL

**Figure 3:**
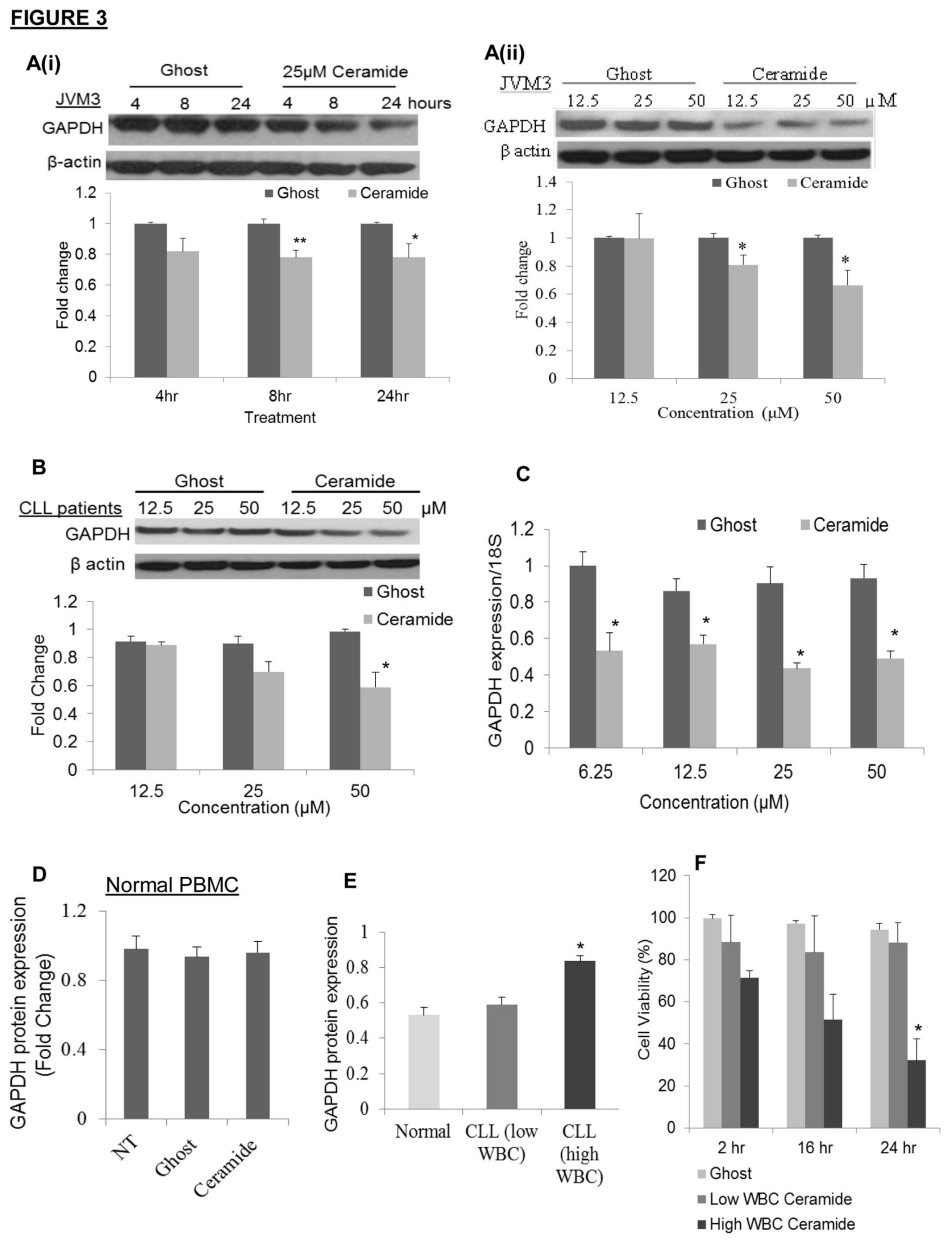
C6-ceramide nanoliposomes target GAPDH in CLL. A). JVM3 cells were treated with (i) 25µM of ghost or C6-ceramide nanoliposomes for varying times, (ii) varying doses of ghost or C6-ceramide nanoliposomes for 24 hours, then Western Blot analysis was performed for GAPDH. Densitometry analysis from replicate experiments (n=4) was performed via ImageJ software. In Figure A(ii) the representative blot demonstrates a decrease in GAPDH at 12.5µM C6-ceramide, a result not observed in the other two distinct replicate experiments. * *P* < 0.05; ** *P <0 .005*. B). CLL patient cells (n=3, analyzed individually) were treated with different doses of ghost or C6-ceramide nanoliposomes for 24 hours, then Western Blot analysis was performed for GAPDH. * *P* < 0.05. The blot represents the effect of C6-ceramide nanoliposomes on one patient sample, however, the graph is an average of the effect on all three patient cells. C). JVM3 cells were treated with different doses of ghost and C6-ceramide nanoliposomes for 24 hours, then qRT-PCR analysis was performed for expression of GAPDH mRNA. Expression was normalized to 18S. * *P* < 0.05. D). PBMC from normal donors (n=3) were treated with 25 µM ghost or C6-ceramide nanoliposome for 24 hours and then Western Blot analysis was performed for GAPDH. E). Basal protein expression of GAPDH was determined via Western Blot analysis on PBMC isolated from normal donors (n=8) or from CLL patients with either a lower WBC count (n=9) or higher WBC count (n=5). Patients with WBC counts >50,000 cells/µL were identified as patients with high WBC count. * *P* < 0.05. F). CLL patient cells with either a lower WBC count (n=4) or higher WBC count (n=3) were treated with 25µM ghost or C6-ceramide nanoliposomes for 2, 16 and 24 hours then an MTT assay was performed * *P* < 0.05.

There is growing evidence suggesting a link between increased dependency on glucose metabolism and overexpression of glycolytic enzymes in cancer cells. Moreover, GAPDH has been shown to be upregulated in many cancers and is the primary target of some chemotherapeutic drugs [30]. Recently, GAPDH has been implicated in promoting cellular survival, chemotherapy-resistance and protection from caspase-independent cell death [31,32]. We now demonstrate that treatment of JVM3 cells with nanoliposomal C6-ceramide leads to a significant decrease in GAPDH protein expression (Figure 3A). There was a significant decrease in GAPDH protein following 8 hours treatment with 25µM nanoliposomal C6-ceramide (Figure 3A(i)).Treatment with varying concentrations of nanoliposomal C6-ceramide for 24 hours significantly decreased GAPDH protein expression (Figure 3A(ii)). Based upon these preliminary studies, we chose to evaluate the physiological and therapeutic consequences of C6-ceramide-mediated GAPDH reduction at 24 hours as a function of concentration. It was also apparent that GAPDH protein expression was decreased following treatment with C6-ceramide nanoliposomes in primary CLL cells (Figure 3B). In addition, using qRT-PCR analysis it was determined that mRNA expression of GAPDH in JVM3 cells was decreased after treatment with nanoliposomal C6-ceramide (Figure 3C). Treatment with nanoliposomal C6-ceramide did not decrease GAPDH protein expression in non-transformed cells from normal donors (Figure 3D), which again alludes to the preferential specificity of nanoliposomal C6-ceramide treatment for cancer cells. Reduction in GAPDH protein preceded induction of cell death. GAPDH reduction was observed as early as 8 hours after treatment with nanoliposomal C6-ceramide, whereas C6-ceramide nanoliposomes induced cell death 12 hours after treatment (data not shown). This indicated that C6-ceramide-induced decrease in GAPDH played a critical role to induce cell death 

 A variety of glycolytic genes are ubiquitously overexpressed in human cancers and are thought to contribute to enhancement of glycolysis [13]. The differential expression of glycolytic enzymes in CLL was previously unknown, so we sought to investigate the expression of GAPDH in CLL. The basal protein level of GAPDH was compared between normal donor PBMC (n=8) and PBMC isolated from CLL patients (n=14). CLL patients were further stratified according to their white blood count (WBC) count at the time their plasma was collected. Patients with WBC counts >50,000 cells/µL were categorized as having high WBC count (n=5), while patients with counts <50,000 cells/µL were in the low WBC count group (n=9). It was evident that GAPDH protein expression was significantly overexpressed in the CLL patient population with high WBC count (Figure 3E). Moreover, treatment with 25µM C6-ceramide nanoliposomes was significantly more cytotoxic in the subset of CLL patients with the higher WBC count (Figure 3F). Collectively, our data suggest that nanoliposomal C6-ceramide is targeting GAPDH and presents a new mechanism for which ceramide is selectively targeting cancer cells. 

### C6-ceramide targets GAPDH-dependent glycolysis

**Figure 4:**
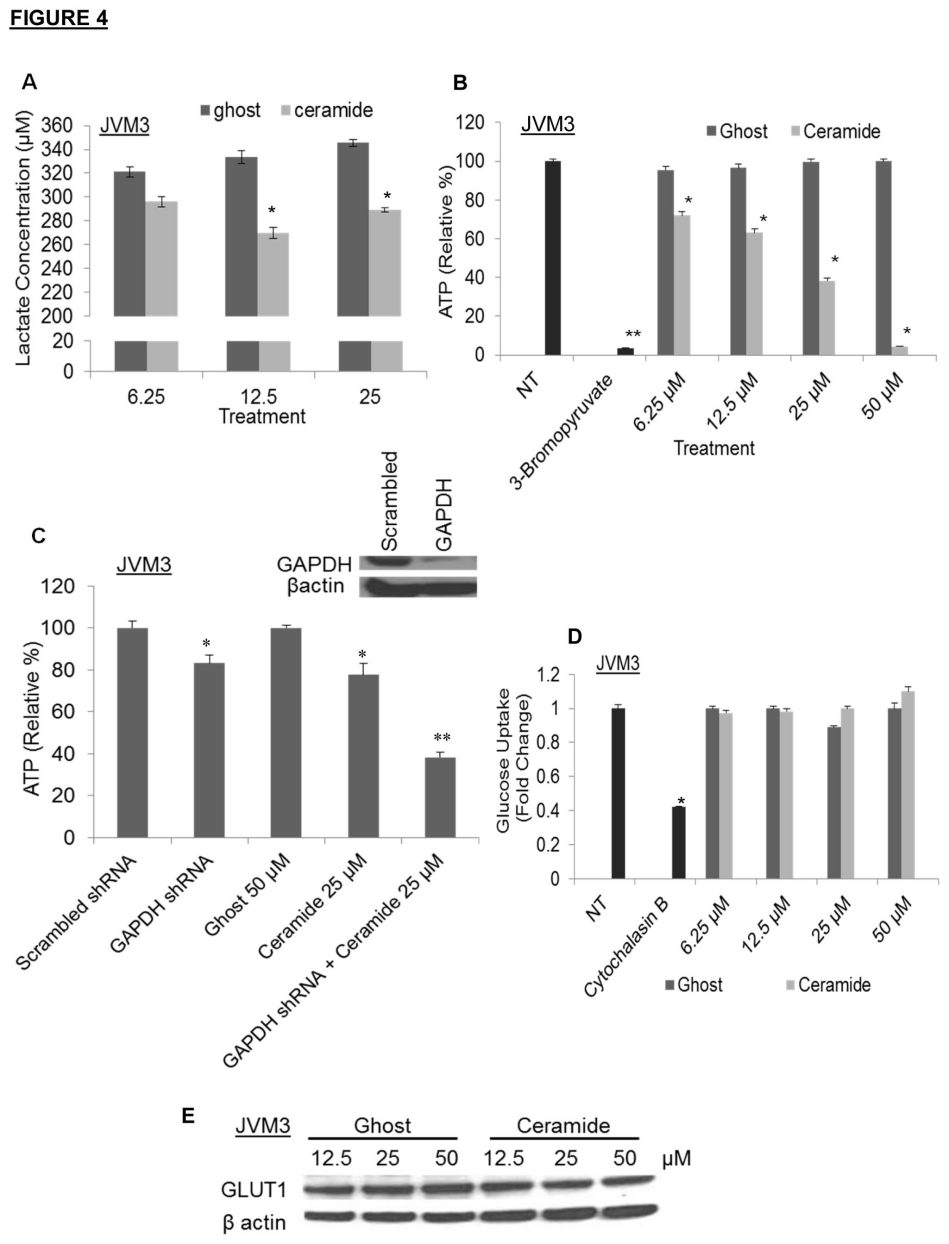
C6-ceramide targets the glycolytic pathway. JVM3 cells were treated with varying doses of ghost or C6-ceramide nanoliposomes for 24 hours, then A) lactate production was analyzed, B) ATP production was analyzed; * *P* < 0.05. The graphs depict average results from three independent experiments. C). GAPDH was effectively knocked down in JVM3 cells via a lentiviral shRNA approach (inset). ATP production was then assessed after 24 hours of treatment with 50µM of ghost or 25µM of C6-ceramide nanoliposomes; * *P* < 0.05; ** *P* < 0.005. D). Glucose uptake was assessed after JVM3 cells were treated with ghost and C6-ceramide nanoliposomes for 24 hours. Cytochalasin B was used as a positive control. E). JVM3 cells were treated with ghost or C6-ceramide nanoliposomes for 24 hours, then Western Blot analysis was performed for GLUT1.

To further demonstrate that C6-ceramide was targeting the glycolytic pathway, consistent with our observation of a decrease in GAPDH expression, we investigated the effects of C6-ceramide upon lactate and ATP production. Treatment with varying doses of nanoliposomal C6-ceramide for 24 hours caused a significant decrease in lactate concentration (Figure 4A) and a dose-dependent decrease in ATP production (Figure 4B) in JVM3 cells. 3-bromopyruvate, a glycolytic inhibitor was used as a positive control in the ATP production experiment. To demonstrate that decreased ATP production was dependent upon ceramide-reduced GAPDH expression we utilized a lentiviral approach to decrease GAPDH in JVM3 cells (Figure 4C inset). We observed a comparable decrease in ATP production between JVM3 cells treated with GAPDH shRNA and 25 µM C6-ceramide nanoliposomal treated JVM3 cells. C6-ceramide nanoliposomal treatment further reduced ATP production in GAPDH-silenced JVM3 cells (Figure 4C). Taken together, these results suggest that ceramide decreased ATP production and it may be partially GAPDH-dependent. 

 Increasing levels of intracellular ceramide correlates with downregulation of nutrient transporters and leads to cellular starvation [10,27]. We wanted to determine if ceramide was also targeting glucose transport in addition to targeting the GAPDH glycolytic enzyme. Results from a tritiated glucose uptake assay showed no significant difference between JVM3 cells treated with ghost or C6-ceramide nanoliposomes (Figure 4D). A significant decrease was observed, however, in cells treated with cytochalasin B, a potent inhibitor of glucose transport. Moreover, protein expression of the glucose transporter, GLUT1, was not altered after treatment with nanoliposomal C6-ceramide (Figure 4E). Further investigation into other glycolytic enzymes, like lactate dehydrogenase (LDH) and pyruvate kinase M2 (PKM2), revealed no difference between the ghost- and C6-ceramide nanoliposome treated groups (data not shown). 

**Figure 5:**
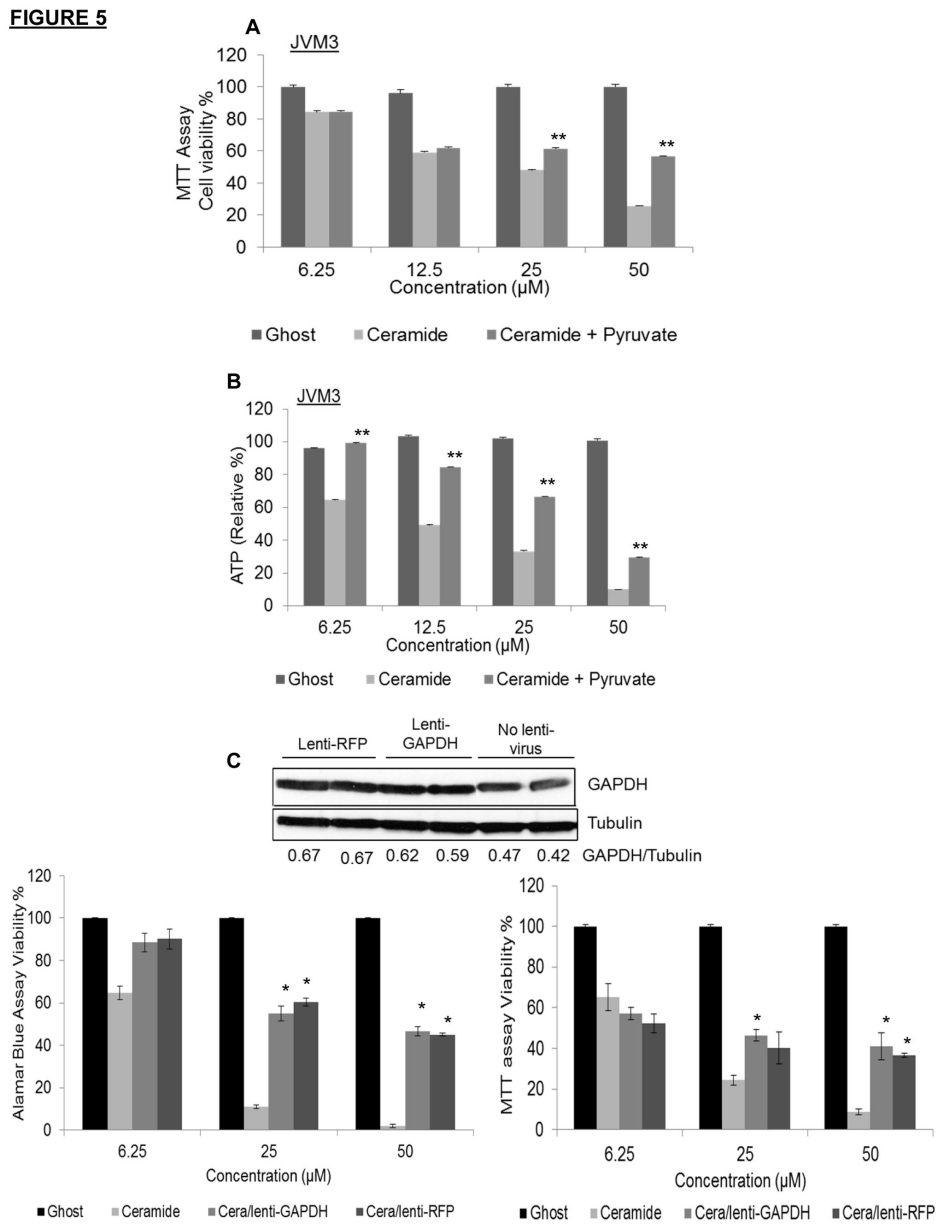
Pharmacological and molecular confirmation that nanoliposomal C6-ceramide targets the glycolytic pathway at the level of GAPDH. A). JVM3 cells were pre-treated for 2 hours with 10mM pyruvate, then treated with ghost or C6-ceramide nanoliposomes for 24 hours. MTT cell viability assay was performed. B). ATP production in these cells was also determined; ** *P* < 0.005. C). JVM3 cells were transduced with lentiviral particles overexpressing GAPDH or viral particles expressing RFP (control). Post transduction, Western blotting analysis was done to determine levels of GAPDH in experimental and control cells (insert). Cells were treated with ghost or C6-ceramide nanoliposomes for 24 hours. Cell viability was determined using MTT assay and alamarBlue assay; * *P* < 0.005.

To illustrate that cell death induced by nanoliposomal C6-ceramide was dependent on targeting of the glycolytic pathway, we pre-treated JVM3 cells with pyruvate, the ultimate downstream product of glycolysis and determined its effect on cell viability and ATP production. Pre-treatment of cells with 10 mM pyruvate led to a significant rescue of cell viability (Figure 5A) and ATP production (Figure 5B) after C6-ceramide nanoliposome treatment. To further demonstrate that reduction in GAPDH protein played a critical role in C6-ceramide-induced cell death, we overexpressed GAPDH in JVM3 cells using a lentiviral transduction overexpression system (Figure 5C). We showed by Western blotting that transduction with lentiviral particles containing the GAPDH gene (Lenti-GAPDH) resulted in overexpression of GAPDH protein (Figure 5C insert). Surprisingly, the procedure itself (control lentiviral particles containing RFP gene, Lenti-RFP) also elevated GAPDH protein expression (Figure 5C insert). In both cases, nanoliposomal C6-ceramide was less effective in inducing cell death when GAPDH protein was elevated. Taken together, overexpression of GAPDH significantly rescued cell death in JVM3 cells after treatment with nanoliposomal C6-ceramide (Figure 5C). 

### Nanoliposomal C6-ceramide displays anti-leukemic effect in CLL animal model

**Figure 6:**
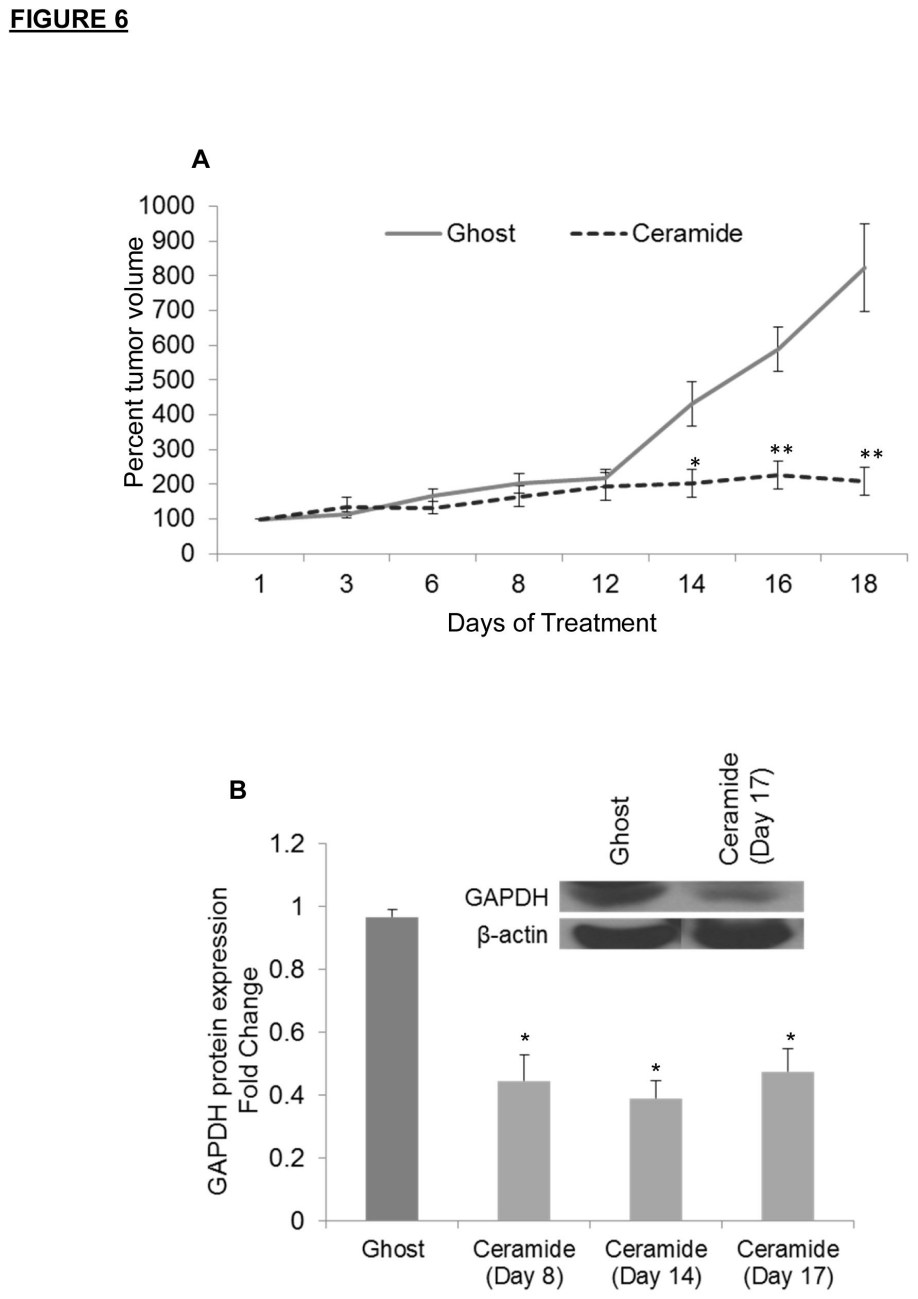
Nanoliposomal C6-ceramide displays anti-leukemic effect in a CLL animal model. A). Two weeks after ten million JVM3 cells were inoculated in the right flank of female Balb/c Nu/nu mice, animals were treated with 40 mg/kg ghost (n=8) or C6-ceramide (n=8) nanoliposomes via tail vein injection. Treatment regimen was every other day over a three week period of time. Tumor size was assessed every other day; * *P* < 0.05; ** *P* < 0.005. B). Leukemic mice following an identical dose regimen were sacrificed on day 8 (n=5), day 14 (n=6) and day 17 (n=6) of C6-ceramide treatment and on day 17 for ghost treatment. Immunoblot analysis for GAPDH protein expression was performed on tumor tissues. A representative blot from day 17 is shown (Figure 6B inset); * *P* < 0.05.

Given that we have demonstrated selective induction of cell death utilizing nanoliposomal ceramide in CLL cells *in vitro*, we next investigated this therapeutic approach in an *in vivo* model. Moreover, this model would allow us to confirm the selective actions of ceramide upon GAPDH-dependent glycolysis *in vivo*. Loisel et al. have reported the establishment of a novel human CLL like xenograft model in nude mice using JVM3 cells [33]. We evaluated the ability of C6-ceramide nanoliposomes to inhibit tumor growth of leukemic CLL cells in xenografts in this immunodeficient mouse model. C6-ceramide nanoliposomes significantly decreased tumor growth beginning on day 13 of treatment (Figure 6A). In addition, C6-ceramide treated tumors did not progress for the remainder of the study. Immunoblot analysis showed that C6-ceramide, but not ghost nanoliposomes, significantly reduced GAPDH protein expression at day 8, 14 and 17 (Figure 6B). ImageJ densitometry analysis for multiple animals is shown, as well as a representative blot from Day 17 (Figure 6B inset). 

Collectively, these results indicate that bioactive ceramide analogues can be incorporated into pegylated nanoliposomal formulations and elicit potent anti-leukemic efficacy in a mouse model of CLL. In addition, these results substantiate our previous *in vitro* data that cell death induction after treatment with C6-ceramide nanoliposomes involves targeting of GAPDH and the glycolytic pathway in CLL. 

## Discussion

The present study identifies dysregulated glucose metabolism as a novel target in CLL. Furthermore, we found that treatment with C6-ceramide nanoliposomes led to preferential induction of caspase-independent, necrotic cell death in CLL cells *in vitro*. Several investigations support a caspase-independent, necroptotic cell death mechanism for chemotherapeutics (tamoxifen, fludarabine) that generate endogenous ceramide [18,34]. In addition, it has been reported that short chain ceramides induce necrotic cell death independent of caspase 3 activation in B cell lymphomas [35]. Several groups, including ours have studied the metabolism of exogenous short chain ceramides in cancer cells. Exogenous C6-ceramide is metabolized into natural ceramides through de-acylation to yield sphingosine followed by subsequent re-acylation with various fatty acids [36]. Exogenous C6-ceramide is also metabolized to C6-sphingomyelin, C6-glucosylcreamide and cerebrosides [36,37,38]. The predominant metabolic pathway of exogenous C6-ceramide is specific to the cancer cell type and concentration of C6-ceramide delivered to the cells. 

We observed that treatment of CLL cells with nanoliposomal C6-ceramide decreased protein and mRNA expression of GAPDH and based upon initial experiments (Figure 3A) we chose to evaluate the physiological consequences of ceramide-mediated decrease in GAPDH at 24 hours as a function of various concentrations of ceramide. GAPDH is known to mediate glycolysis and is responsible for oxidizing glyceraldehyde-3-phosphate to 1,3-biphosphoglycerate. Until recently, GAPDH has been considered as a constitutive housekeeping gene and was used as a control for normalizing changes in expression of protein and genes. Current studies suggest that GAPDH is upregulated in many cancers and is the primary target of some chemotherapeutic drugs [30]. The tumor suppressor TP53 has been shown to increase GAPDH expression in endothelial cells [39] and significant increases in GAPDH expression were also observed in breast cancer cells stimulated with growth factors [40]. Interestingly, GAPDH has been shown to play a key role in opposing caspase-independent cell death and promoting cellular survival [31]. It has been suggested that GAPDH overexpression may assist cancer cells in evading apoptosis and cell death mechanisms [30]. This protection from cell death is mediated by an elevation in glycolysis and enhanced ATP levels [32]. Moreover, it has been demonstrated that overexpression of GAPDH is sufficient to protect chronic myelogenous leukemia (CML) cells from imatinib-induced caspase-independent cell death and knockdown of GAPDH resensitizes cells to imatinib-induced cell death [41]. Therefore, combining a drug that reduces GAPDH expression (like ceramide) with imatinib could prove to have therapeutic benefit in overcoming this drug resistance. 

In addition to its role in the glycolytic pathway, GAPDH has also been shown to serve other functions in the cell which remain to be elucidated. Although generally in a cytosolic form, the GAPDH protein can also be found in the nucleus and nuclear functions of GAPDH include transcriptional regulation, DNA repair, and maintenance of telomere structure [42]. There is rapid shortening of telomere length when cells are treated with exogenous, short-chain ceramides [43]. Ogretmen and colleagues demonstrated that overexpression of GAPDH results in protection of telomeric DNA in response to exogenous ceramide treatment [43]. Further analysis showed that ceramide was inhibiting nuclear localization of GAPDH and showed that a potential mechanism of ceramide-mediated shortening of telomeres in somatic cells leads to cell senescence while maintenance of telomeres is associated with immortality of cancer cells. This suggests another mechanism by which increased GAPDH expression contributes to a survival advantage in cancer cells. 

Elevation of glucose uptake and glycolysis in cancer cells can depend heavily on the upregulation of glucose transporters [44]. Others have shown that nutrient transporter down-regulation is critical in ceramide-induced cell death [10], so we sought to clarify this hypothesis in CLL. Using a radiolabeled glucose uptake assay, we demonstrated that the ability of ceramide to induce cell death in CLL was not due to blocking nutrient transport of glucose into the cell. In addition, expression of the GLUT1 glucose receptor was not regulated by ceramide. This lack of effect upon nutrient transport further suggests direct effects upon glycolytic enzymes overexpressed in cancer. 

We identified GAPDH as being overexpressed in a population of CLL patients. In addition to the Western blot analysis, we also re-analyzed previous microarray data from CLL patients [45] using Gene Expression Omnibus (GEO) to investigate dysregulation of glycolytic enzymes in CLL. Interestingly, we found that all of the enzymes in the glycolytic pathway were upregulated in the CLL patients with a poor prognosis (data not shown). Based on our results and the independent microarray data, we conclude that glycolysis appears to be upregulated in a subset of CLL patients with either higher white blood cell counts and/or a more aggressive clinical course. 

Our findings demonstrate that the cytotoxicity of C6-ceramide is due to targeting of glucose metabolism in CLL. Previous studies reported that inhibitors of ATP production, like bromopyruvate, effectively induce cell death in cancer [46]. The abolishment of cellular ATP production proves effective due to the increased dependence on glycolysis and ATP production in oncogenic cells. Moreover, treatment with koningic acid, an inhibitor of GAPDH selectively induces cell death in cancer via ATP deprivation [47]. In a similar fashion, we also show that nanoliposomal C6-ceramide treatment induces cell death in CLL through targeting of the glycolytic pathway and results in decreased ATP and lactate production. Even though our studies indicate that shRNA knock down of GAPDH reduced ATP production similar to 25 µM C6-ceramide nanoliposomal treatment, the fact that ceramide augmented this shRNA approach suggests that ceramide may be targeting the Warburg effect through both decreased GAPDH as well as an undefined secondary mechanism. 

To better understand and confirm if inhibition of the glycolytic pathway was the means by which ceramide was inducing cell death in CLL, we carried out experiments that involved pre-treatment with a final end product of glycolysis. It was determined that pre-treatment of CLL cells with pyruvate was sufficient to rescue ATP depletion and cell death that was otherwise induced after treatment with the C6-ceramide nanoliposome. In addition, we transiently overexpressed GAPDH in CLL cells to demonstrate rescue of C6-ceramide-induced cell death in these cells. Interestingly, we also observed rescue of cell death in control cells transduced with control viral particles. Yet, protein analysis showed that both, the experimental and control cells overexpressed GAPDH. This alludes to the fact that transient overexpression of GAPDH partially protects CLL cells from C6-ceramide-induced cell death. 

 Confirming *in vitro* studies, we provide clear evidence that nanoliposomal C6-ceramide demonstrated *in vivo* efficacy via tumor growth inhibition in a murine xenograft model of CLL. We are aware of a recently described mouse model for CLL generated by co-injection of primary human CLL cells and T cells into NOD/ SCID mice, but have chosen to focus our studies on the mouse xenograft model because we can obtain tissue for *ex vivo* Western analysis of GAPDH protein levels [48]. In our *in vivo* studies we observed that treatment with nanoliposomal C6-ceramide effectively decreased tumor burden without systemic side effects. This is consistent with previous studies from our laboratory and others which show that this C6-ceramide nanoliposome formulation results in tumor regression in animal models of cancer and hematological malignancies [6,9,22]. In addition, it was expected that this nanoliposomal formulation would be relatively non-toxic to the animals, as it was previously selected by the Nanotechnology Characterization Laboratory (National Cancer Institute) for extensive toxicology and stability testing. (Detailed information on the toxicology studies of the “Ceramide Liposomes” can be found at http://ncl.cancer.gov/working_technical_reports.asp). *In vivo* studies also confirm that ceramide is targeting GAPDH in CLL, as protein isolated from tumor tissue showed an overall decrease in GAPDH expression following treatment with C6-ceramide nanoliposomes. 

In conclusion, we show C6-ceramide nanoliposomes preferentially inhibit the altered metabolism of glucose in leukemic cells via downregulation of GAPDH, resulting in induction of necrotic cell death. We provide the first evidence that GAPDH is overexpressed in a subset of CLL patients. Our findings provide a metabolic explanation for the increased sensitivity and selectivity of cancer cells to ceramide. Taken together, these results suggest that nanoliposomal C6-ceramide could be an effective novel therapy for patients whose cancer cells overexpress GAPDH, including those with CLL. 
